# Incidence, Outcomes, and Prediction of Postoperative Urinary Retention After a Nonurologic Procedure

**DOI:** 10.5435/JAAOSGlobal-D-19-00149

**Published:** 2020-05-19

**Authors:** Haidar M. Abdul-Muhsin, Nicholas Jakob, Stephen Cha, Nan Zhang, Adam Schwartz, Anojan Navaratnam, Aqsa Khan, Mitchell Humphreys

**Affiliations:** From the Department of Urology (Dr. Abdul-Muhsin, Jakob, Dr. Navaratnam, Dr. Khan, and Dr. Humphreys), Department of Research Biostatistics (Dr. Cha and Dr. Zhang), and the Department of Orthopedic Surgery (Dr. Schwartz), Mayo Clinic Arizona, Phoenix, AZ.

## Abstract

**Purpose:**

To develop a prognostic model to estimate postoperative urinary retention (POUR) after lower limb arthroplasty.

**Methods:**

One thousand two hundred twenty patients underwent 1,374 joint replacement operations (812 knees and 562 hips) between December 2008 and May 2014. Detailed variables were collected. A multivariable logistic regression model was used to identify the independent predictors for POUR. Boot strapping and stepwise elimination was used to design a predictive nomogram.

**Results:**

There were 124 incidents of POUR (9.02%) in 118 patients (90 knee, 34 hip, *P* = 0.001). On univariate analysis, patients who developed POUR were older (*P* < 0.001), had higher American Association of Anesthesiology scores (P = 0.007), underwent knee replacement (0.001), were obese (body mass index > 35) (*P* = 0.04), and were hypertensive (*P* = 0.029), with a history of benign prostatic hyperplasis (BPH) (*P* < 0.001) or neurologic disorders (*P* = 0.024). On multivariable analysis, age (60 to 69 years, *P* = 0.023, 70 to 79 yrs *P* = 0.008, >80 years *P* = 0.003), knee replacement (*P* = 0.014), and history of BPH (*P* = 0.013) were the independent predictors of POUR. A score was assigned to each predictor (total = 31). The C-index was 0.65. There were three risk categories as follows: 0 to 50, 51 to 85, and 86+ points resulting in 3.3%, 7.2%, and 14.0% risk of retention, respectively.

**Discussion:**

This nomogram reliably predicts the risk of POUR in patients undergoing hip and knee arthroplasties and may help planning preoperative interventions to decrease the risk of this complication.

Postoperative urinary retention (POUR) is common in elderly male patients after various surgical procedures.^1^ The risk of POUR after any procedure is estimated to occur between 4% and 6% in the general surgical population.^2,3^ However, the occurrence of POUR depends on the specific surgical intervention where the incidence is thought to be 20 fold higher after lower limb arthroplasty (LLA).^3^ The reported rates of POUR after LLA are widely variable (0% to 75%), and this variability in the literature stems from the heterogeneity of sample sizes, patient populations, type of anesthesia, postoperative analgesic regimens, and the wide array of definition used to describe POUR in published series.

LLA is one of the most commonly performed procedures in the United States where more than 450,000 procedures were performed in 2014. Per capita utilization of total knee arthroplasty increased by 99% for primary cases and by 106% for revision TKA during the past decade.^4^ The occurrence of POUR in the postoperative period potentially manifests many health- and cost-related consequences such as prolonged hospitalizations, increased costs, and infection-related complications that may threaten prosthetic implantations.^5-7^ Using a national inpatient sample of more than 400,000 patients, Wu et al found that patients who developed urinary retention can be predicted and they may benefit from interventional measures.

There is an abundance of literature that evaluated the potential predictors of POUR. Nevertheless, the results were not consistent, and there is no overall consensus regarding the most important factors predisposing patients to retention and the statistical weight of each predictor. This hinders the efforts to individualize preventive and prophylactic plans. Moreover, most of the studies done looked at this issue from anesthetic and orthopaedic perspective. Little is known regarding the specific urological characteristics that lead to this problem after a nonurologic procedure.

The specific aim of this study was to individually prognosticate POUR after LLA in a large cohort of elderly male patients using a detailed database and to assess the weight of each predictor. The resulting nomogram may allow the individual clinician to counsel and intervene before surgery. Secondary measures examined were the rates of POUR, final urological outcomes, and infectious complications after the occurrence of retention.

## Methods

After the institutional review board approval, all male patients who underwent hip or knee replacement surgery at our institution between December 2008 and May 2014 were included. Patients were identified using a common procedural terminology. There were no exclusion criteria. An extensive and thoroughly annotated database was designed. This covered all potentially related variables and included detailed baseline demographic, past medical and urological medications, and preoperative, intraoperative, anesthetic, and postoperative variables.

Baseline demographic variables included age, marital status, body mass index, and detailed medical, surgical, and urologic histories. Medication history included diuretics, alpha blockers, five alpha reductase inhibitors, phosphodiesterase inhibitors, anticholinergic, narcotics, muscle relaxants, blood thinners, and long-term preoperative antibiotics. Urologic history included the history of bladder outlet obstruction (BPH or urethral stricture disease), any previous urological procedure (classified by type), and past urologic workup for BPH if available (including prostate specific antigen (PSA). prostate volume, uroflowmetry, postvoid residual volume, and urodynamic study), and most recent culture and/or urinalysis. Operative data included type (knee versus hip), laterality, sequence of joint replacement (first, second, and third), American Association of Anesthesiology (ASA) score, operative time (minutes), estimated blood loss in milliliter, type of anesthesia (general, spinal, epidural, and local), and total narcotic usage as measured by total morphine equivalence, time to ambulation, and total length of stay (days).

POUR was defined as the patient's inability to void spontaneously at any point in the postoperative period or after indwelling catheter removal, requiring a urinary catheter reinsertion. The general clinical pathway is to remove the urinary catheter on postoperative day 1 with a voiding trial after distention of the urinary bladder with 500 mL of normal saline and ask the patient to void. If the patient fails to void, we routinely use the bed side ultrasound to evaluate the postvoid residual volume. The decision of urinary catheter reinsertion was mainly made on an independent basis depending on the individual patient history, symptoms, and the provider discretion. Urology consultation was obtained when the patient failed multiple attempts of voiding trials. The choice of catheterization (self-intermittent catheterization versus indwelling catheterization) and duration of catheter insertion was recorded and the need to initiate or increase the dose of BPH medication(s). The final outcome for patients who failed these conservative measures was reported, including the results of postoperative uroflowmetry, urodynamic study, prostate volume (cc), and type of bladder outlet procedure if performed. A separate descriptive analysis was conducted for patients who developed POUR to report any urinary- or joint infection-related complications.

The end point for this study was the occurrence of POUR after any joint replacement surgery. The statistical analysis included univariable and multivariable analyses using a stepwise logistic regression model to identify independent predictors for the abovementioned endpoint (using odds ratio with 95% confidence interval and *P* value ≤0.05). This was followed by the backward elimination methodology to identify the most accurate and parsimonious model with the smallest number of variables to predict the outcomes of interest. For each model, bootstrapping with 1,000 simulations was conducted to randomly selected observations with replacement from our cohort to assign a risk score for each independent predictor. Using the average odds ratios from simulations, a predictor was picked when its *P* value was significant in more than 50% of times, and it was assigned a score that reflected its prognostic effect. A total score was calculated by obtaining the sum of all these products of predictors which then correlated with the probability of POUR. Calibration and assessment of the predictive accuracy of our nomogram was made using a calibration plot. Total scores were lumped together to propose descriptive categories with distinct risks of urinary retention. Statistical analyses were performed by SAS version 6.4 software (SAS institute) and R Version 3.4.4 (R Core Team, 2013). All statistical tests were two-sided, and the level of statistical significance was set at *P* < 0.5.^8,9^

## Results

One thousand two hundred twenty-two patients underwent 1,374 joint replacement operations (812 knee and 562 hip) during the study period. There were 124 incidents of POUR (9.02%) in 118 patients (90 knee, 34 hip, *P* = 0.001). A detailed description of patients' baseline and preoperative variables is shown in Table1. Operative details are shown in Table 2. Of note, 34.8% of the hip patients and 41.3% knee patients did not have an intraoperative catheter. For those who had a catheter, their average durations of catheterization before the voiding trial were 1.4 days (SD 0.55) and 1.6 days (SD = 0.56) for the knee and hip, respectively. Intraoperative catheterization or duration of catheterization was not a statistically significant variable to predict POUR.

**Table 1:** Preoperative Variables

Variable	No. of Available Records	Overall		No		Retention		*P* Value
		(N = 1,374)		(N = 1,250)		(N = 124)		
Age	1,374	69.03	±10.36	68.7	±10.49	72.35	±8.27	<0.001
Age category, n (%)	1,374							0.002
<60 yr	1,374	216	16%	210	97%	6	3%	
60–69 yr	1,374	449	33%	410	91%	39	9%	
70–79 yr	1,374	504	37%	451	89%	53	11%	
>80 yr	1,374	205	15%	179	87%	26	13%	
BMI	1,373	30.84	±5.46	30.9	±5.54	30.25	±4.66	0.21
Most recent PSA (pre-op)	443	1.56	±1.91	1.55	±1.93	1.63	±1.73	0.81
Uroflow performed before joint replacement?	486	0.51	±0.50	0.5	±0.50	0.61	±0.49	0.14
Volume voided (mL)	247	226.99	±151.30	228.17	±154.22	217.37	±127.17	0.73
Maximum flow rate (mL/sec)	244	15.41	±9.90	15.77	±10.14	12.53	±7.15	0.11
Average flow rate (mL/sec)	238	7.86	±4.84	8.08	±4.95	6.16	±3.42	0.05
Postvoid residual (mL)	247	64.44	±106.31	56.71	±101.64	127.48	±123.62	<0.001
History of diabetes, n (%)	1,374	184	13%	162	88%	22	12%	0.14
History of neurologic disorder, n (%)	1,374	159	12%	137	86%	22	14%	0.024
History of bleeding disorder, n (%)	1,374	9	1%	9	100%	0	0%	0.34
History of urologic disorder, n (%)	1,374	649	47%	580	89%	69	11%	0.049
History of hypertension, n (%)	1,374	827	60%	741	90%	86	10%	0.029
History of hyperlipidemia, n (%)	1,374	184	13%	170	92%	14	8%	0.47
Obesity_BMI 35 m, n (%)	1,374	236	17%	223	94%	13	6%	0.038
History of coronary artery disease, n (%)	1,374	222	16%	196	88%	26	12%	0.13
History of BPH, n (%)	1,374	313	23%	268	86%	45	14%	<0.001
History of UTI, n (%)	1,374	1	0%	1	100%	0	0%	0.75
History of urethral stricture disease, n (%)	1,374	10	1%	10	100%	0	0%	0.32
History of urinary retention, n (%)	1,374	15	1%	13	87%	2	13%	0.56
Diuretic usage, n (%)	1,374	294	21%	266	90%	28	10%	0.74
BPH medication usage, n (%)	1,374	303	22%	268	88%	35	12%	0.08
Erectile medication usage, n (%)	1,374	104	8%	96	92%	8	8%	0.62
Anticholinergic medication usage, n (%)	1,374	19	1%	16	84%	3	16%	0.30
Anticoagulant medication usage, n (%)	1,374	930	68%	844	91%	86	9%	0.68
Antibiotic usage, n (%)	1,374	115	8%	108	94%	7	6%	0.25
History of past urologic procedure	1,349	271	20%	248	92%	23	8%	0.69

BMI = body mass index

**Table 2:** Operative Variables

Variable	No. of Available Records	Overall		No		Retention		*P* Value
		(N = 1,374)		(N = 1,250)		(N = 124)		
Duration of hospitalization (d)	1,370	2.85	±0.93	2.83	±0.91	3.05	±1.04	0.012
MED	1,374	442.19	±428.79	436.96	±432.76	494.83	±384.14	0.15
Location, n (%)	1,374							0.001
Knee	1,374	812	59%	722	89%	90	11%	
Hip	1,374	562	41%	528	94%	34	6%	
Sequence, n (%)	1,374							0.96
First	1,374	1,216	89%	1,106	91%	110	9%	
Second	1,374	155	11%	141	91%	14	9%	
Third	1,374	1	0%	1	100%	0	0%	
Fourth	1,374	0	0%	0	100%	0	0%	
Fifth	1,374	2	0%	2	100%	0	0%	
Type of anesthesia, n (%)	1,368							0.51
Local	1,368	58	4%	55	95%	3	5%	
Regional	1,368	6	0%	5	83%	1	17%	
Spinal	1,368	1,296	95%	1,177	91%	119	9%	
General	1,368	8	1%	8	100%	0	0%	
ASA score, n (%)	1,347							0.007
1	1,347	16	1%	15	94%	1	6%	
2	1,347	638	47%	582	91%	56	9%	
3	1,347	690	51%	626	91%	64	9%	
4	1,347	3	0%	1	33%	2	67%	

ASA = American Association of Anesthesiology, MED = Morphine equivalent dose

On univariable analysis, patients who developed POUR were older (*P* < 0.001), had higher ASA score (*P* = 0.007), underwent knee replacement (*P* = 0.001), were less likely to be obese with body mass index > 35 kg/m^2^ (*P* = 0.038), more likely to be hypertensive (*P* = 0.029), with a history of BPH (*P* < 0.001) or neurologic disorders (*P* = 0.024). In patients who underwent urological workup before surgery, the average flow rate and postvoid residual on uroflowmetry assessment were different among the two groups (*P* = 0.05 and *P* < 0.001). Regarding outcomes, most of the patients did not require a bladder outlet procedure (110 no procedure, 4 TURP, 2 HoLEP, and 2 urethral dilations). Urinary retention did not result in increased risk of postoperative infective joint complications or affected the duration of hospitalization.

**Figure 1:**
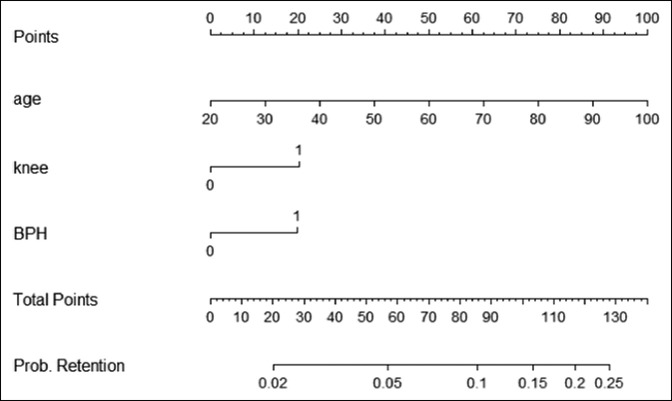
Figure demonstrating the predictive nomogram to predict postoperative urinary retention after weight-bearing joint replacement surgery.

**Figure 2:**
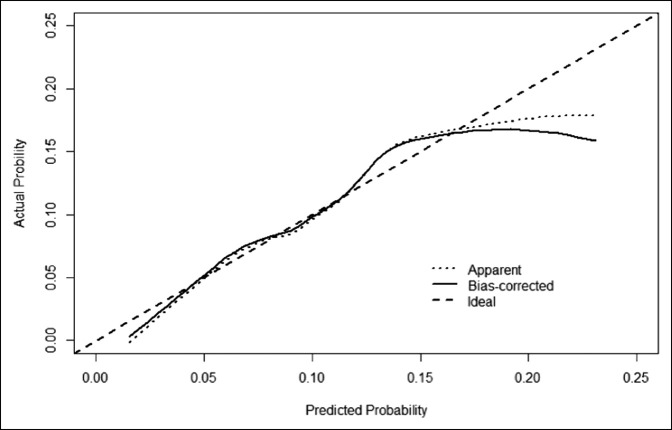
Figure demonstrating the calibration plot to assess the accuracy of urinary retention nomogram after joint replacement surgery. The “Ideal” line represents perfect prediction because the predicted probabilities equal the observed probabilities. The “Apparent” line represents the calibration using our sample. The “Bias Corrected” line is derived via resampling. The closer the bias corrected line to the ideal line the more accurate is the prediction within that specific range. When the line is below the ideal line, it is overprediciting the outcome and when it is above the ideal line, it is underpredicting the outcome.

On multivariable analysis, age (60 to 69 years, *P* = 0.023, 70 to 79 years *P* = 0.008, >80 years *P* = 0.003), knee replacement (*P* = 0.014), and history of BPH (*P* = 0.013) were the independent predictors of postoperative retention (Table 3). A score was assigned to each predictor. A total score was used to develop a predictive nomogram based on these predictors (total score = 140 point), as shown in Figure 1. The C-index of our nomogram was 0.65. The application of this nomogram resulted in three risk categories, depending on the total score with 0 to 50, 51 to 85, and 86 + points, resulting in 3.3%, 7.2%, and 14% average risk of retention, respectively. The calibration plot is shown in Figure 2.

**Table 3:** Multivariable Analysis and A Final Step Down Model to Predict Postoperative Urinary Retention

Variable	Odds Ratio Estimate	Lower 95% Confidence Limit for Odds Ratio	Upper 95% Confidence Limit for Odds Ratio	*p* value	c_stat
N = 1,322 retention 122					
Age, yr					
60–69	2.805	1.154	6.821	0.023	
70–79	3.187	1.302	7.803	0.011	
>80	3.955	1.52	10.287	0.005	
Knee	1.757	1.151	2.681	0.009	
Obesity (BMI > 35)	0.487	0.253	0.938	0.031	
History of neurologic disorder	1.529	0.915	2.557	0.105	
History of hypertension	1.36	0.894	2.07	0.151	
History of BPH	1.627	1.041	2.542	0.033	
ASA 3 and 4	0.925	0.611	1.399	0.711	
History of diabetes	1.438	0.858	2.408	0.168	
History of UTI	0.767	<0.001	>999.999	1.000	
History of urethral stricture disease	<0.001	<0.001	>999.999	0.985	
History of urinary retention	0.986	0.206	4.716	0.986	
History of BPH medication	0.985	0.607	1.599	0.953	
History of erectile dysfunction medication	0.88	0.408	1.898	0.745	
History of anticoagulant medication	2.239	0.603	8.319	0.229	
History of urologic surgery	0.727	0.438	1.207	0.218	0.68
Step down model					
Age, yr					
60–69	2.79	1.154	6.745	0.023	
70–79	3.292	1.374	7.886	0.008	
>80	4.099	1.629	10.316	0.003	
Knee replacement	1.687	1.112	2.56	0.014	
BPH	1.67	1.116	2.5	0.013	0.65

ASA = American Association of Anesthesiology, BMI = body mass index, UTI = urinary tract infection

## Discussion

Our results demonstrated a 9% incidence of POUR in a large cohort of male patients undergoing LLA. Using a cohort that largely represents the average patient population who undergo this procedure, we were able to identify multiple predictors including age, type of prosthesis, and history of BPH and ASA score. We were able to design an accurate predictive tool that may help individualize the risk of POUR using readily available preoperative factors that does not require a dedicated preoperative specialty evaluation and can be easily obtained using preoperative screening. An internal validation using the boot strap technique was possible, and the discriminative ability of this nomogram as measured by its C-index could reliably differentiate among three distinct categories of POUR with clinically meaningful differences.

POUR after LLA has been thoroughly evaluated in the past by multiple investigators since the introduction of this procedure, given the established relationship between the risk of prosthetic infection and POUR, resulting in prolonged catheterization. Early investigators proposed that evaluation and treatment of lower urinary tract symptoms should take place before accepting patients for arthroplasty because of the unacceptable high risks of retention and subsequent infection.^5^ Subsequently, the exponential increase in the utilization of joint replacement procedure was paralleled by a paradigm shift in the management of this problem with high focus on the identification of the risk factors and prevention. Baldini et al and Balderi et al proposed algorithms for early detection, prevention, and management of POUR that highly depend on the risk factors of developing retention.^3,10^ However, the identification of these risk factors was based on the review of previous studies with widely variable definitions of POUR and lack of testing of some important urological factors.

The different types, routes of administration of anesthesia, and postoperative analgesics has received extensive attention and demonstrated conflicting results.^3,10^ The effect of general anesthesia on the lower urinary tract function and micturition reflex is well established.^11,12^ However, the effect of spinal and epidural anesthesia on bladder control is quick and can last to up to 8 hours after anesthesia.^13^ Nevertheless, the comparison of anesthetic modalities in this specific context is not well understood and is difficult to assess because of the conflicting results.^1,2,10,14,15,16,17,18,19,20,21,22,23^ Bladeri et al concluded that patients who received epidural or systemic anesthesia had similar risks of POUR after an extensive systemic review of the pertinent literature which is consistent with our results where no statistically significant differences in the modality of anesthesia used were identified.^10^ Of note, we used the morphine equivalent dose as a collective measure to assess the impact of this effect on POUR.

Many previously conducted studies identified predictive factors that were similar to the ones identified in this study such as increasing age and history of BPH.^18,21,24-26^ Other factors that were assessed included the intraoperative fluid volume and operative time. Certain studies suggested that intravenous fluid volume and bladder volume at certain levels were independent risk factors for POUR,^20,27^ whereas, other studies concluded that intravenous fluid volume was a mere reflection of operative time and association with retention was not confirmed.^28^ This study did not find an association between operative times and POUR which contrasts previous findings that showed that longer operations lead to higher risk of POUR.^29,30^ This may be explained by the similar ranges in operative time for all of the arthroplasty cases in our cohort, which creates difficulty in accurately detecting outcome differences based on this factor.

For preoperative urologic evaluation, the only identified risk factor was a history of BPH. When a preoperative urological workup was available, there was no correlation between various uroflowmetry, urodynamic parameters, prostate volume evaluation, or the previous administration of BPH medications. We mainly relied on objective measures and preoperative testing rather than the patient-filled preoperative questionnaires such as the International Prostate Symptom Score; the role of which remains controversial. Postoperatively, most of our patients did not require extensive workup because POUR resolved spontaneously with no need for a bladder outlet procedure in most cases. Although bacteriruria is an established risk that is directly related to the duration of catheterization and subsequent joint infection, we did not identify an increased risk in infectious joint complications in our cohort.^5-7^

Our study has many strengths including using a large representative sample of patients with multiple urological and nonurological variables that were not previously tested. We were able to individualize the risks of POUR using a nomogram that may pave the road for prospective pharmacologic intervention in selected high-risk patients. Our nomogram can provide a practical clinical guide to calculate the risk of urinary retention. To use the nomogram (Figure 1), each risk factor will have an individual score by aligning that to the top line. After that the total score is calculated by adding the sum of the individual scores and aligning that to the probability of retention. For example, a 60-year-old man undergoing a knee replacement with no history of prostatic enlargement will have a total score of 70 (age = 50 points, knee = 20, and BPH history = 0). This corresponds to a medium risk category 51 to 85 and a respective risk of retention of 7.5%, whereas a 70-year-old man with a previous history of BPH undergoing the same procedure will have total score of 102.5 (age = 62.5, knee = 20, and BPH history = 20) and corresponding high risk of 14.8%. This significant difference in risk of POUR can lead the surgeon to properly counsel the patient preoperatively and to consider a urological evaluation before that.

Our study was performed in a tertiary center with high LLA volume performed by multiple orthopaedic surgeons with standardized perioperative pathway and rehabilitation regimen. However, the results have to be evaluated in light of the retrospective nature of the study and the general limitations of any predictive nomogram. Given the fact that the entire patient cohort in this study presented to our hospital, the study is subjected to a selection bias. Moreover, it is essential to highlight the fact that the c-index of any nomogram can assess its discriminative ability (the ability to differentiate the risk of two patients), and not the ability to compare the incidences of predicted and actual event. The latter is preferably tested using external prospective validation. Finally, a few variables which were examined occurred at low rates, limiting our ability to accurately analyze those in a multivariate analysis model.

This is a large study to analyze the incidence, risk factors, and outcomes of urinary retention after LLA. This has led to many important conclusions. Importantly, the statistical weight of what we established as risk factors was revealed. Most of these outcomes are inherently related to patient characteristics and not the intraoperative or postoperative course. Finally, the nature of this problem is transient in most patients and does not require immediate surgical intervention. Collectively, we think that the results of this study will provide a more objective tool to counsel patients and to plan prophylactic measures to prevent the occurrence of this adverse outcome.
